# Supplementary figures and images for: Optimization of a tri-drug treatment against lung cancer using orthogonal design in preclinical studies
Source: PeerJ. 2023 Jul 10;11:e15672. doi: 10.7717/peerj.15672 (PMC10340110; doi:10.7717/peerj.15672)

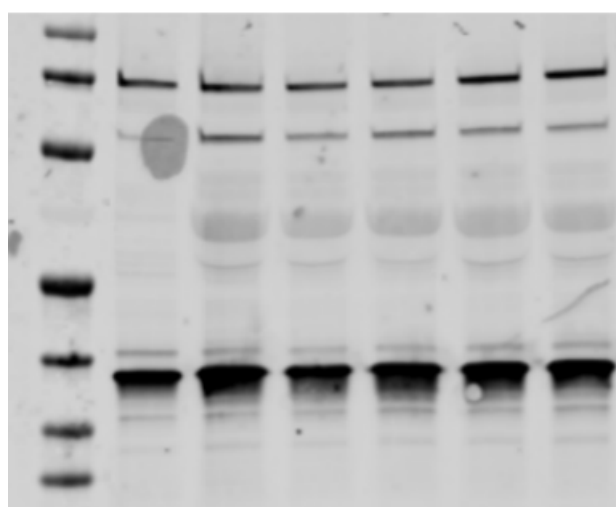

Supplement: Supplemental Information 1 [file peerj-11-15672-s001.pdf]

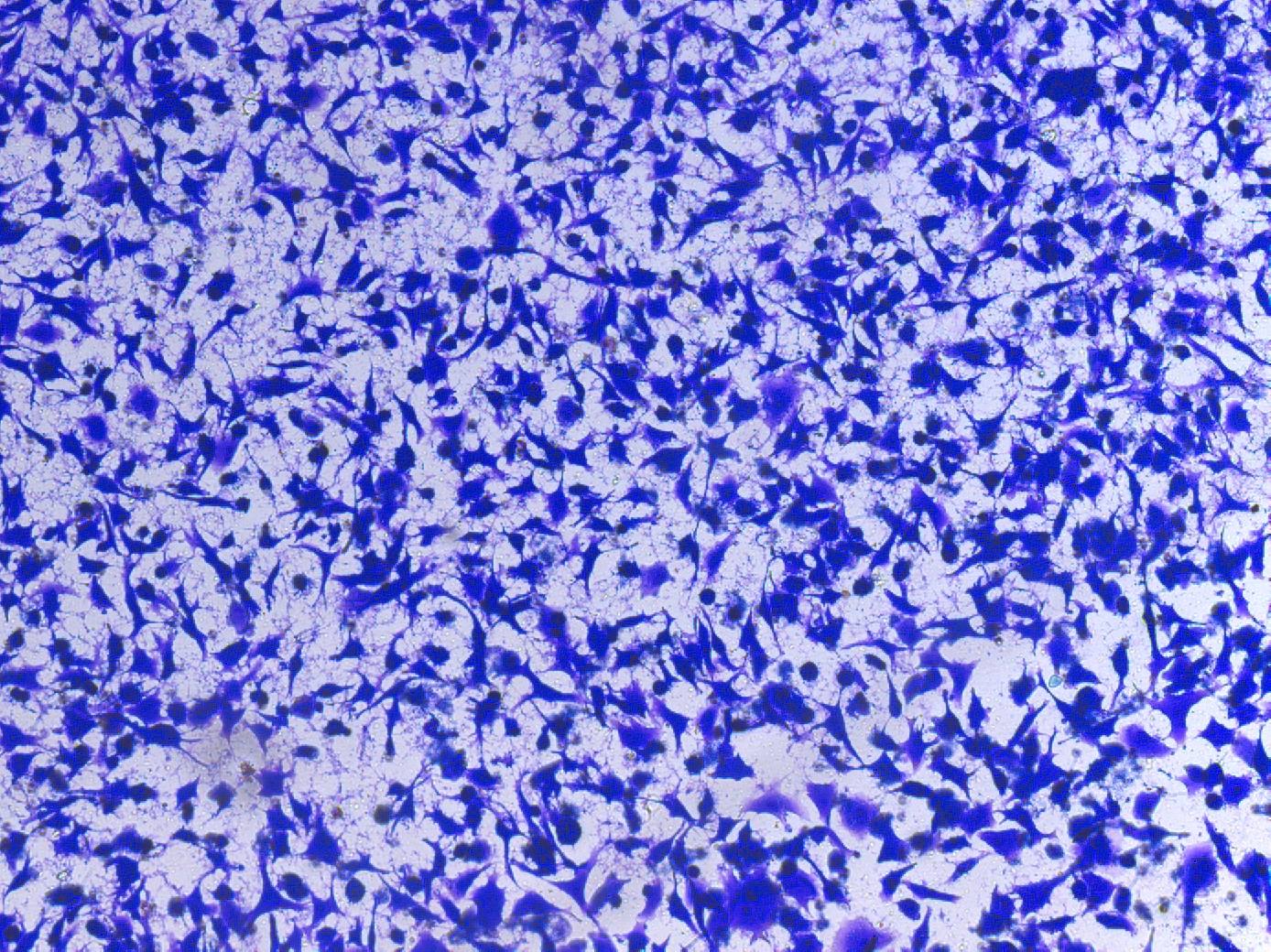

Supplement: Supplemental Information 2 [file peerj-11-15672-s002.zip › Invasion/ctrl-1.jpg]

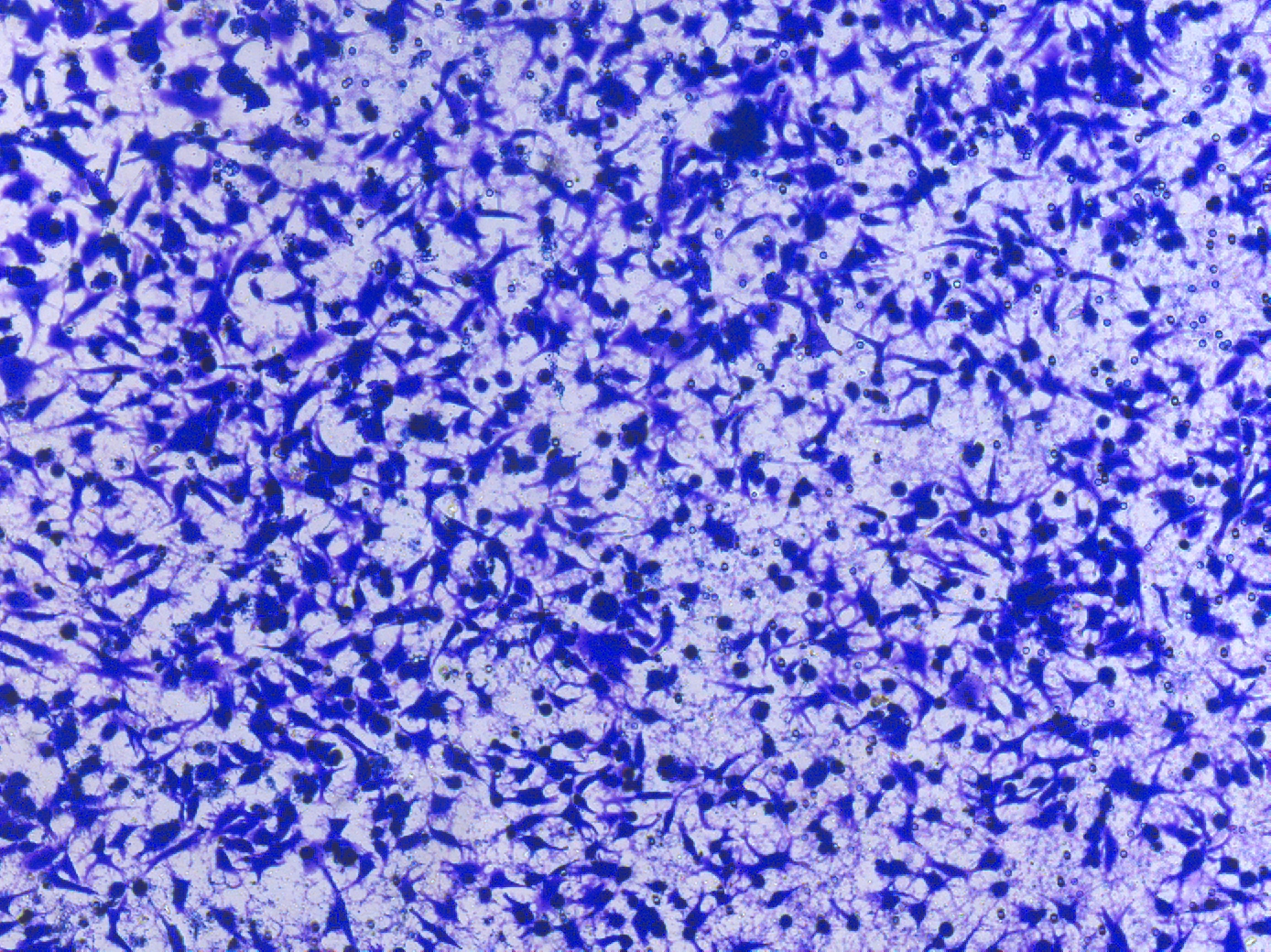

Supplement: Supplemental Information 2 [file peerj-11-15672-s002.zip › Invasion/ctrl-2.jpg]

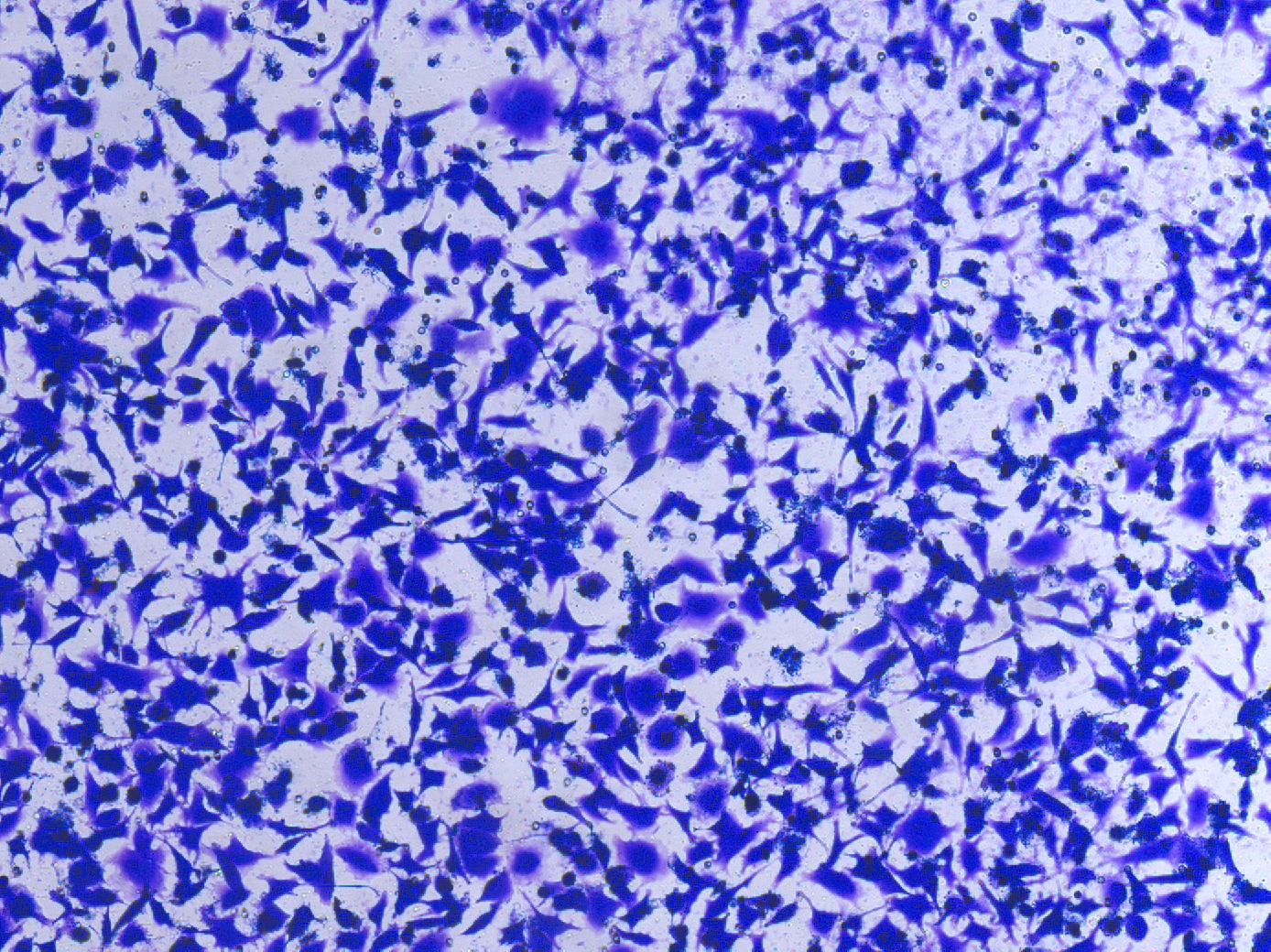

Supplement: Supplemental Information 2 [file peerj-11-15672-s002.zip › Invasion/ctrl-3.jpg]

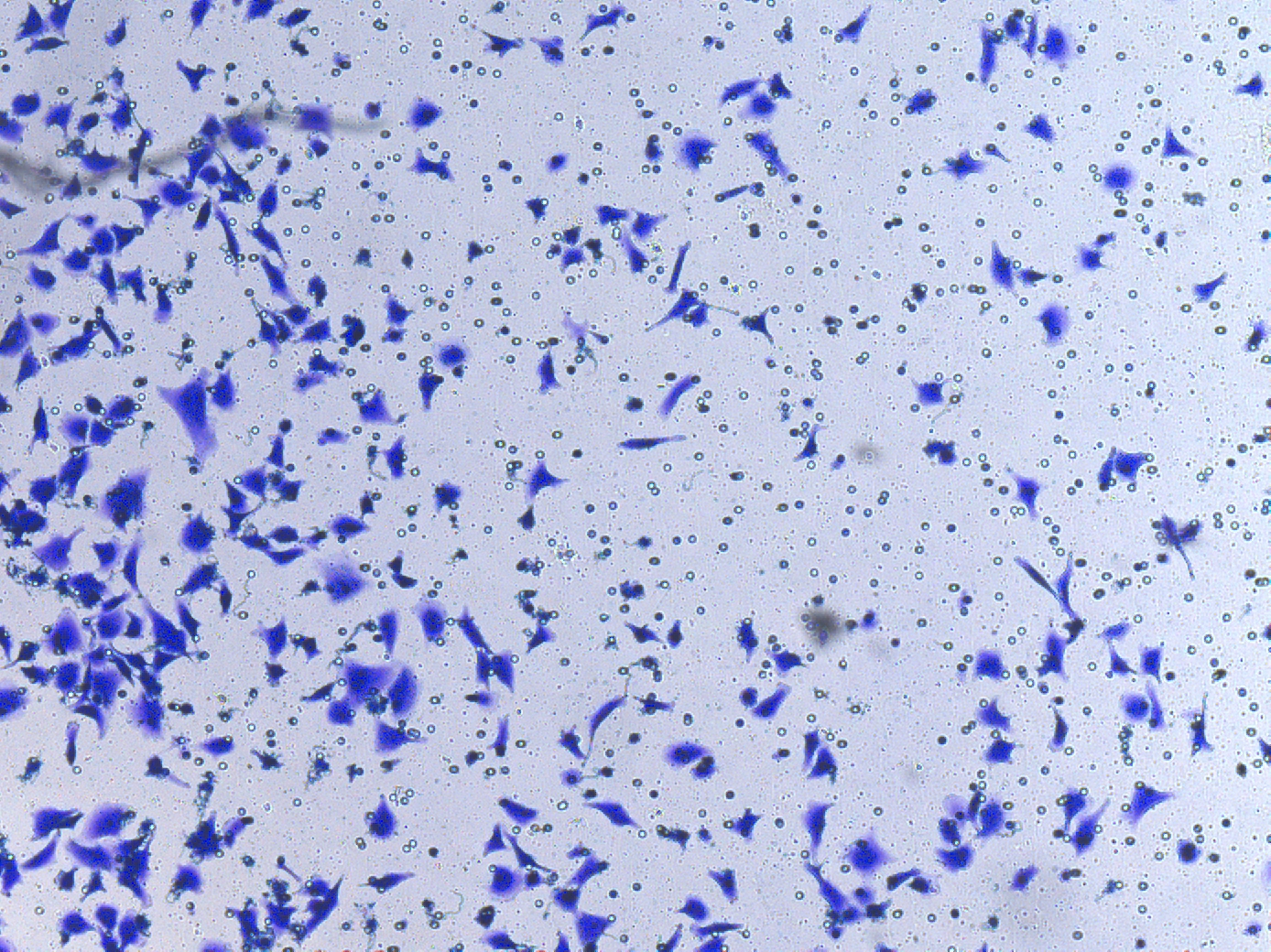

Supplement: Supplemental Information 2 [file peerj-11-15672-s002.zip › Invasion/PSR-1.jpg]

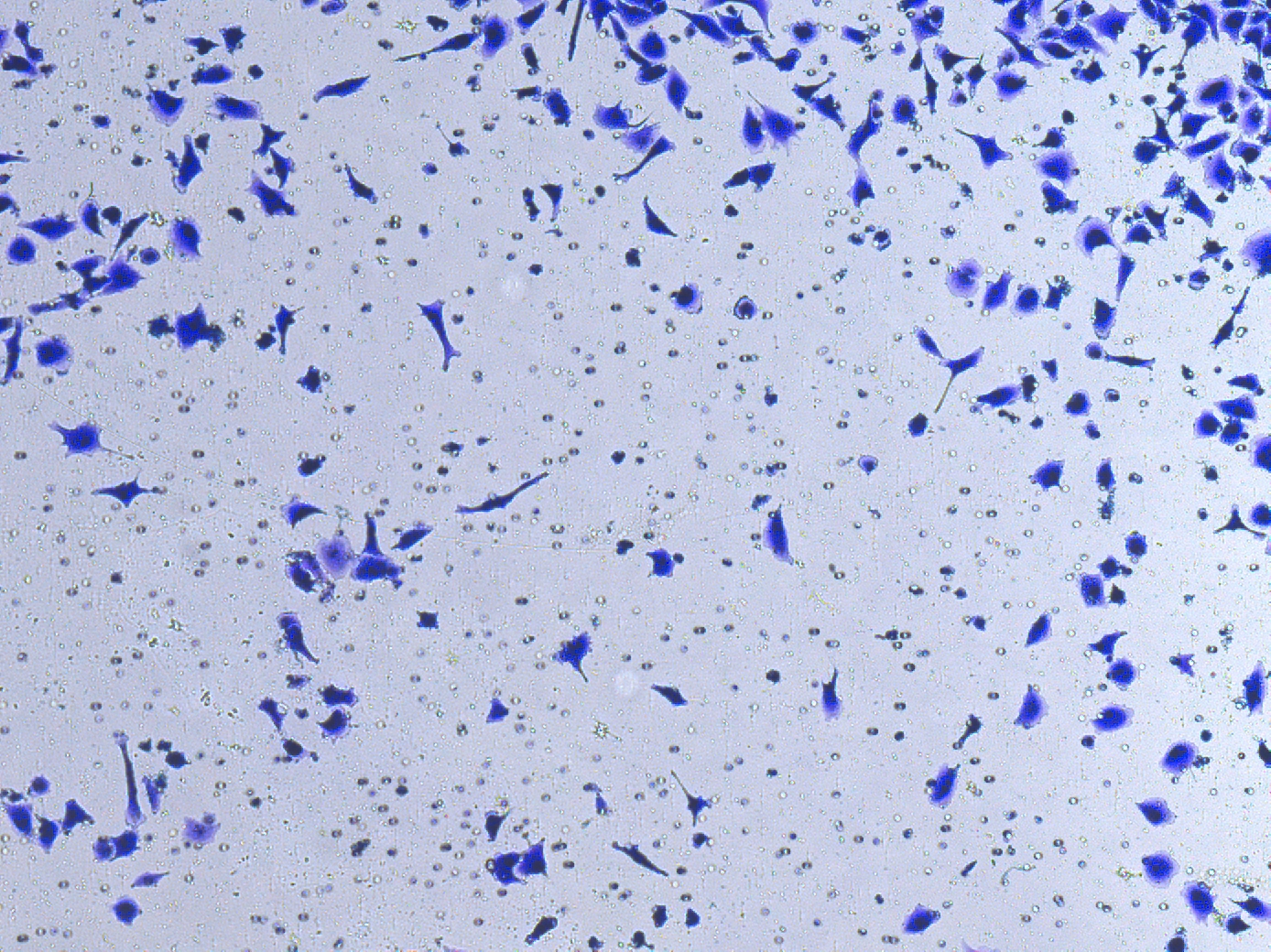

Supplement: Supplemental Information 2 [file peerj-11-15672-s002.zip › Invasion/PSR-2.jpg]

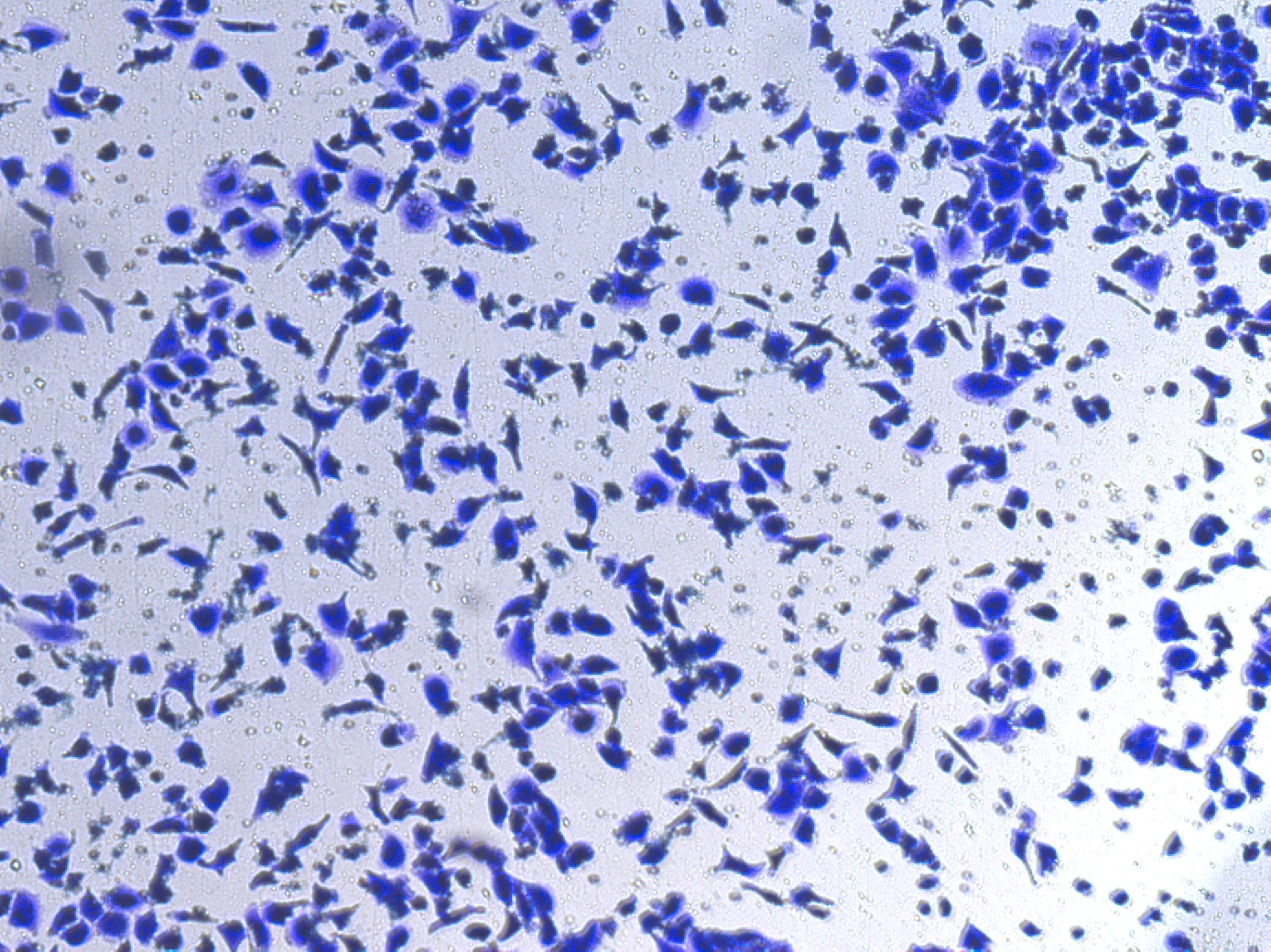

Supplement: Supplemental Information 2 [file peerj-11-15672-s002.zip › Invasion/PSR-3.jpg]

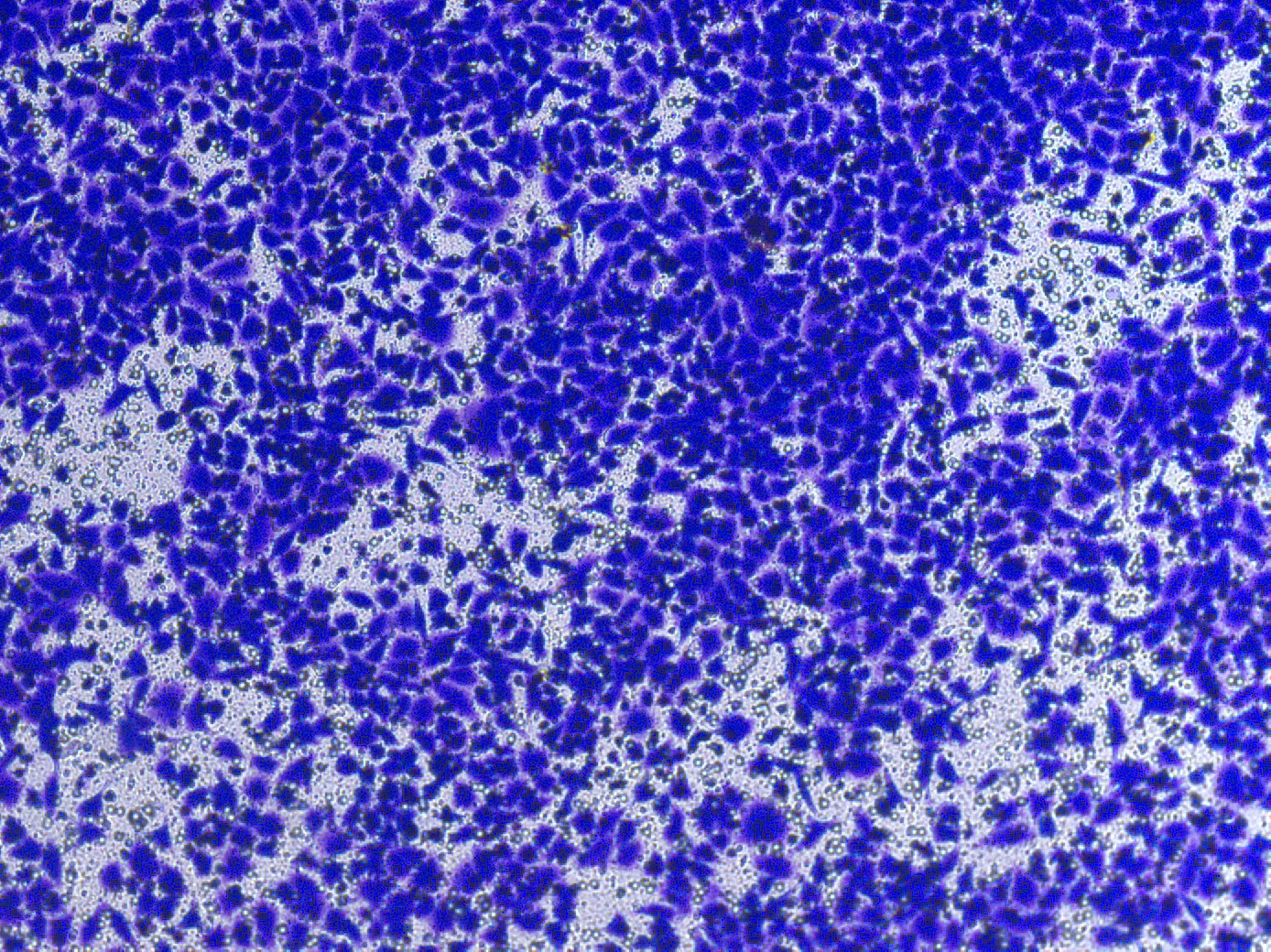

Supplement: Supplemental Information 2 [file peerj-11-15672-s002.zip › migration/ctrl-1.jpg]

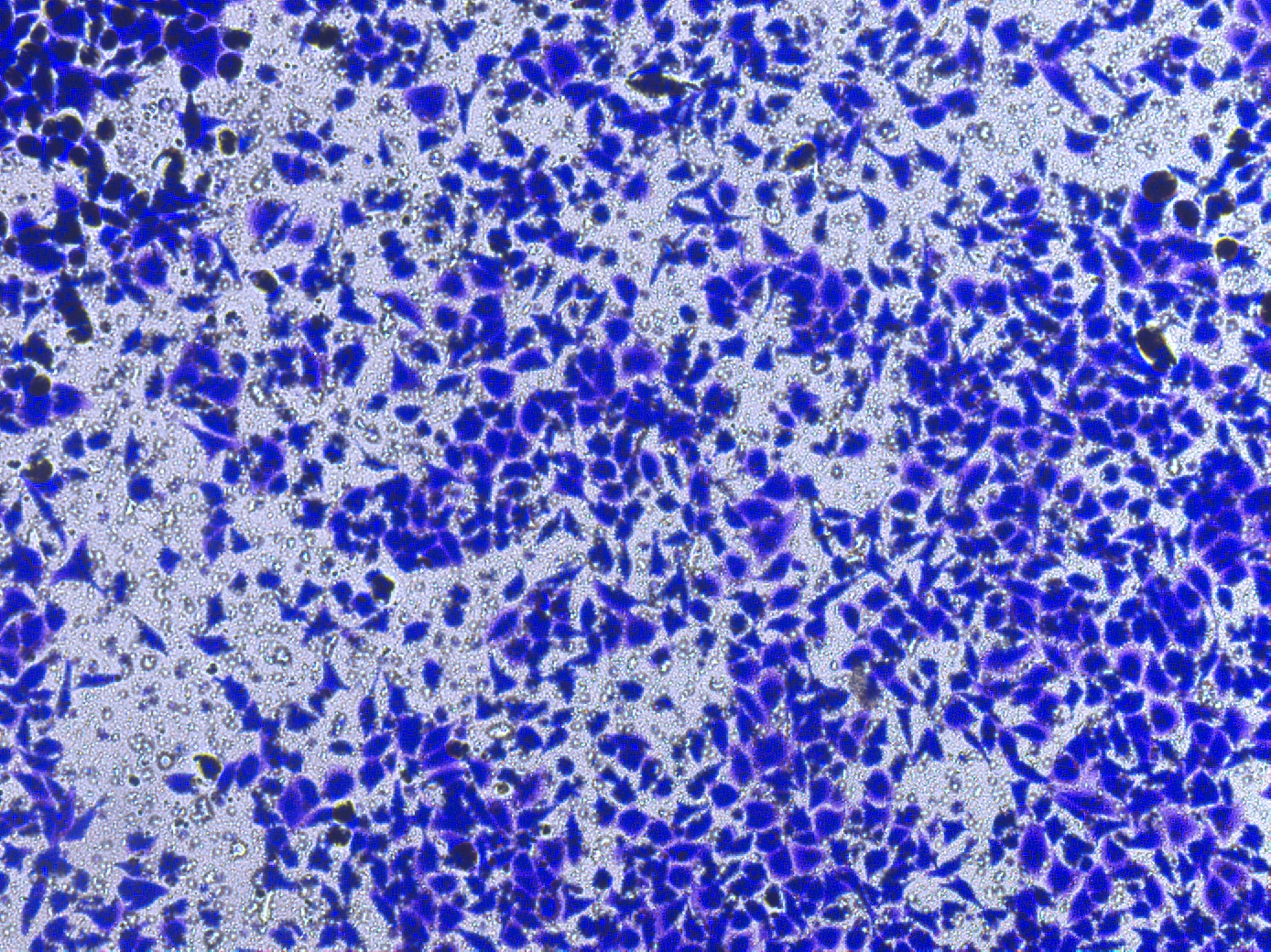

Supplement: Supplemental Information 2 [file peerj-11-15672-s002.zip › migration/ctrl-2.jpg]

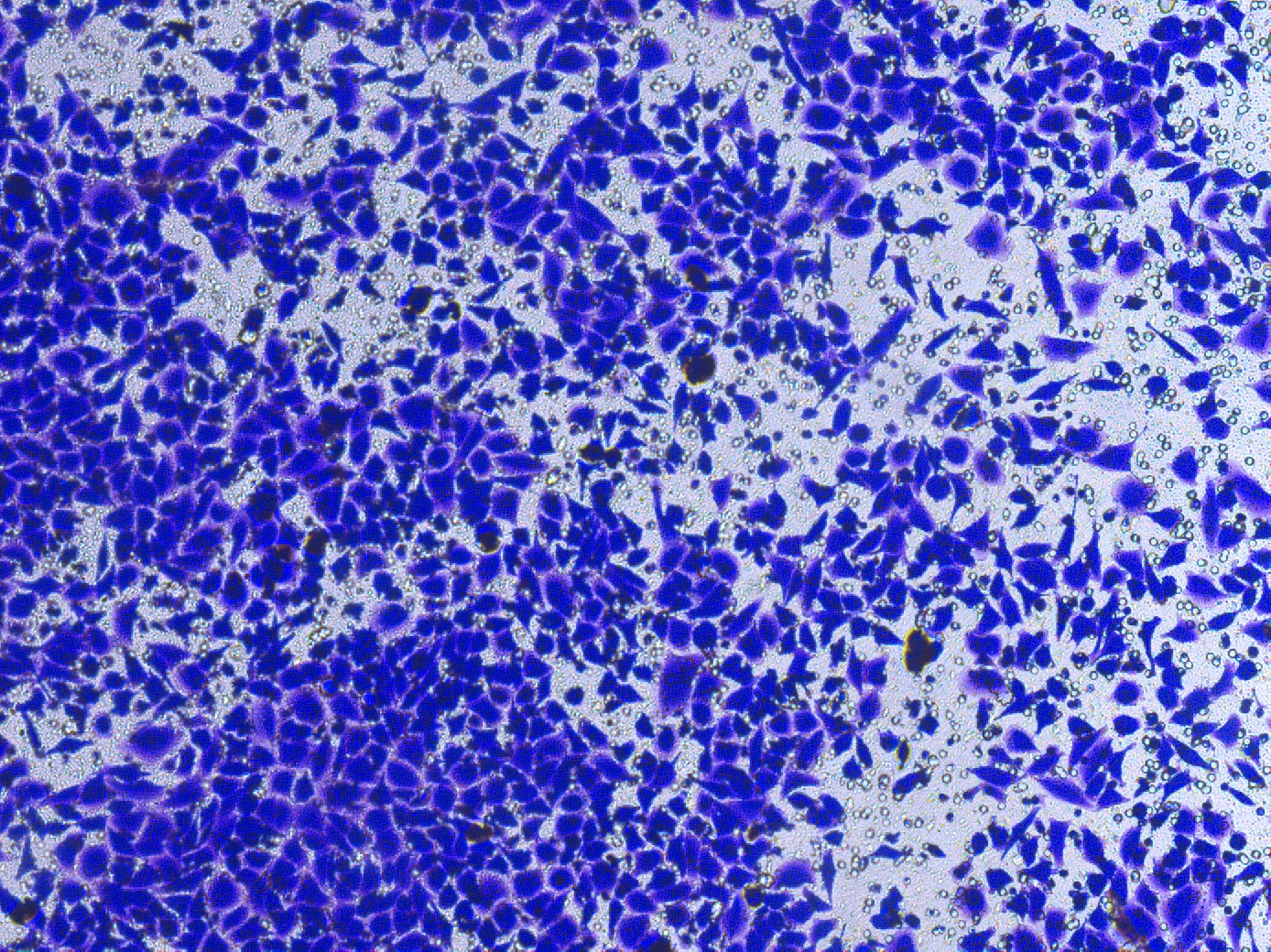

Supplement: Supplemental Information 2 [file peerj-11-15672-s002.zip › migration/ctrl-3.jpg]

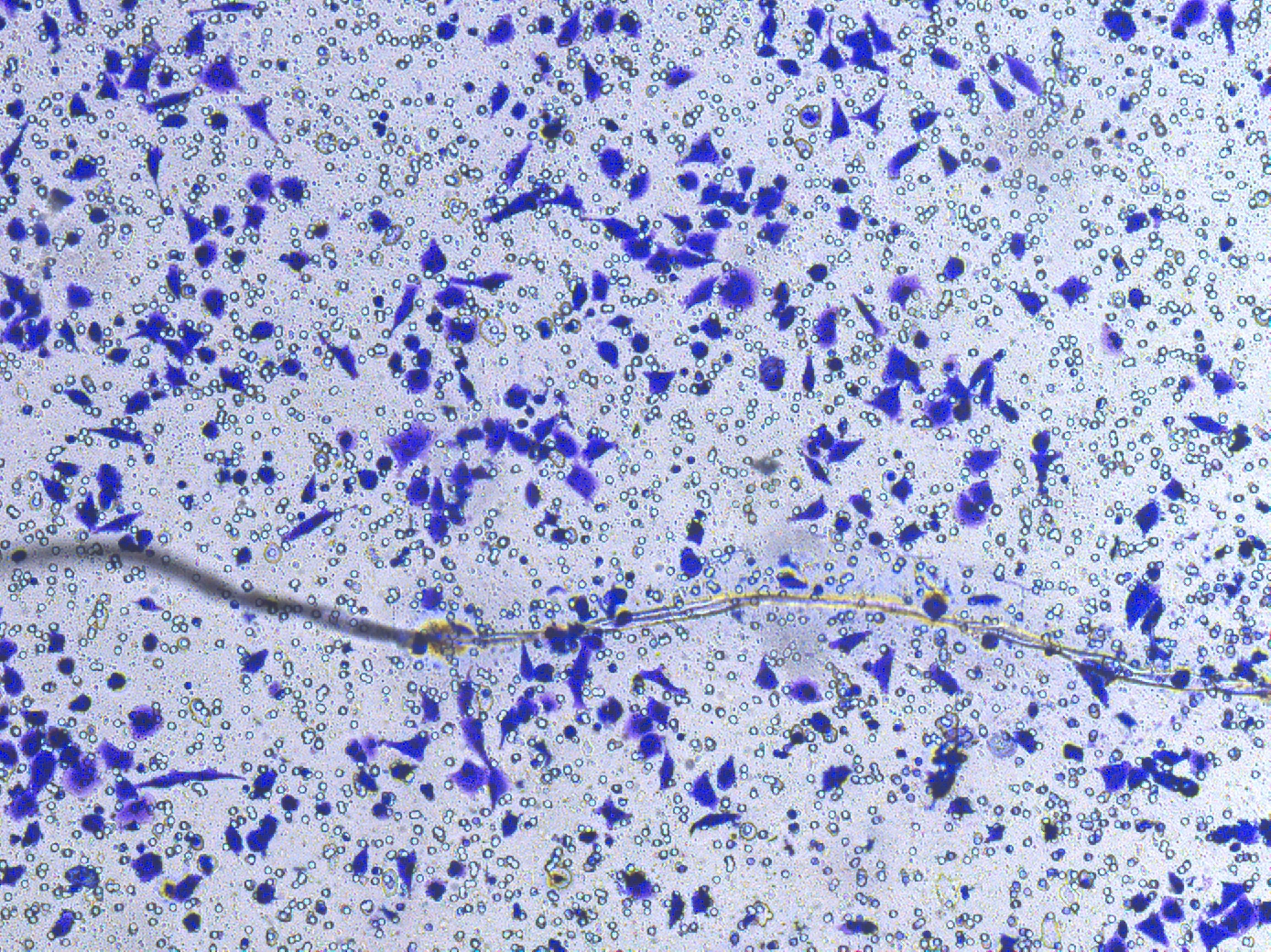

Supplement: Supplemental Information 2 [file peerj-11-15672-s002.zip › migration/PSR-1.jpg]

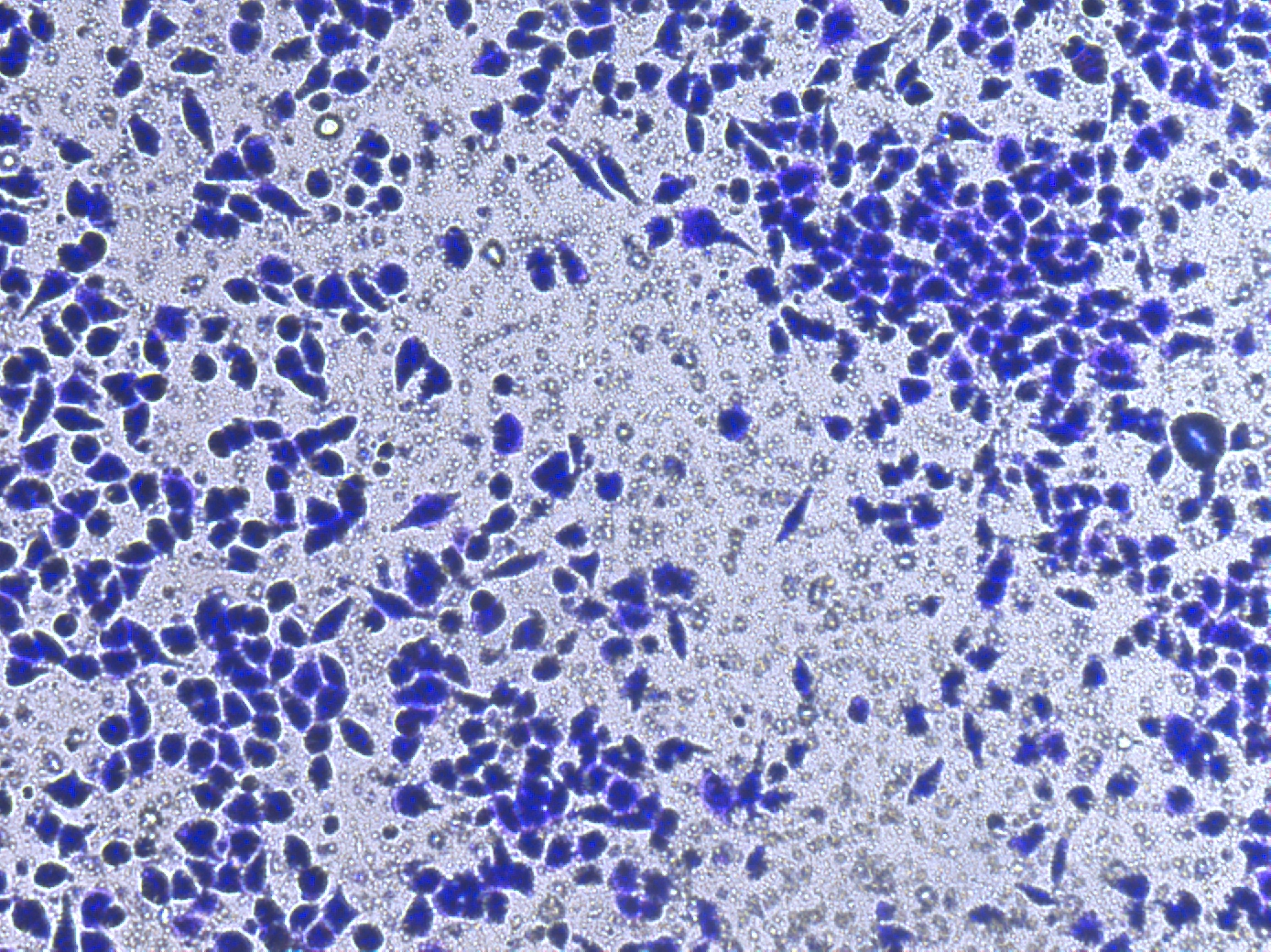

Supplement: Supplemental Information 2 [file peerj-11-15672-s002.zip › migration/PSR-2.jpg]

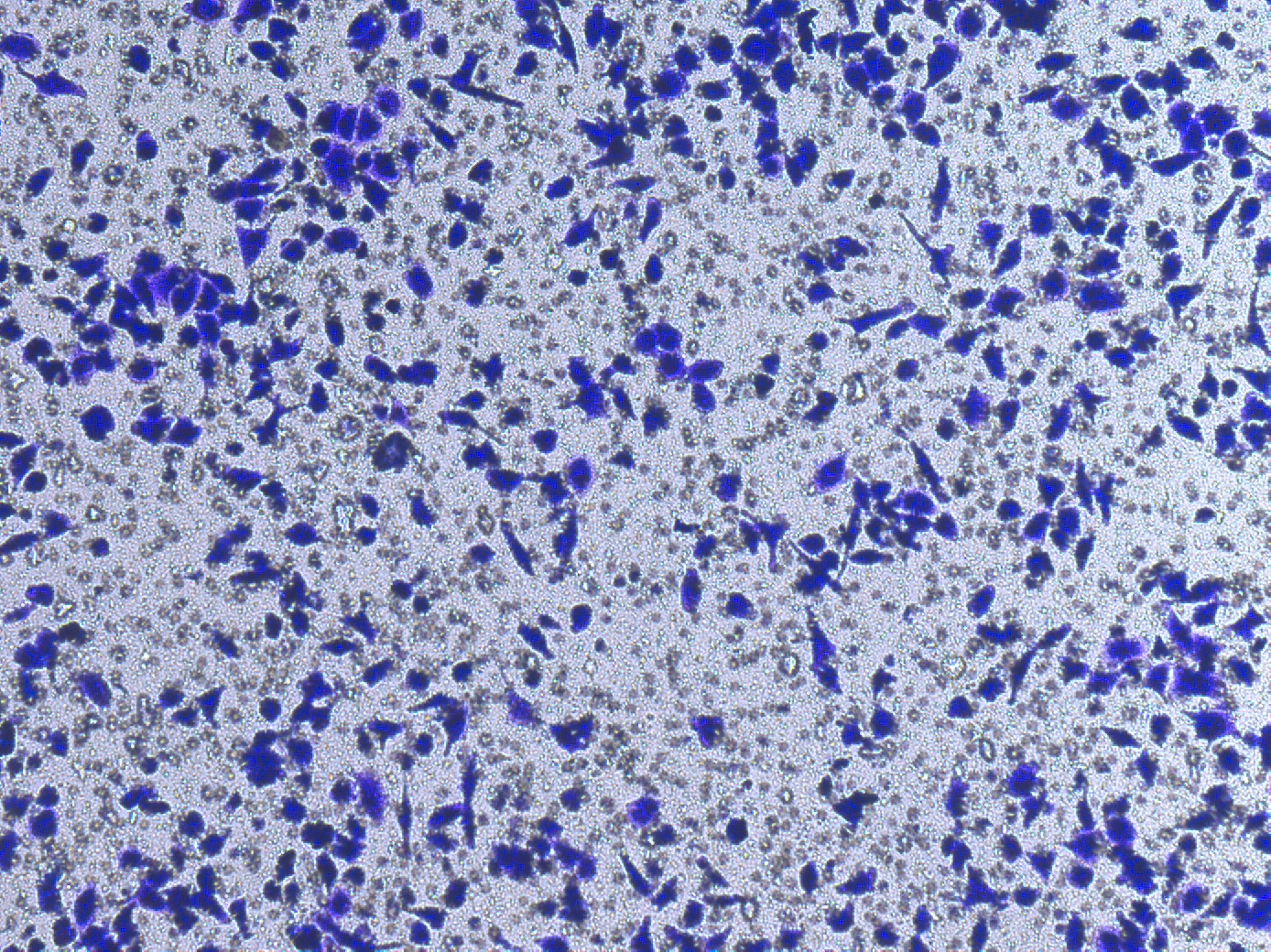

Supplement: Supplemental Information 2 [file peerj-11-15672-s002.zip › migration/PSR-3.jpg]
